# A study on the radiomic correlation between CBCT and pCT scans based on modified 3D-RUnet image segmentation

**DOI:** 10.3389/fonc.2024.1301710

**Published:** 2024-02-22

**Authors:** Yanjuan Yu, Guanglu Gao, Xiang Gao, Zongkai Zhang, Yipeng He, Liwan Shi, Zheng Kang

**Affiliations:** ^1^ College of Electronic Engineering, Zhangzhou Institute of Technology, Zhangzhou, Fujian, China; ^2^ Department of Radiation Oncology, the First Affiliated Hospital of Xiamen University, Xiamen, Fujian, China

**Keywords:** radiomics, cone-beam CT, rectal cancer, 3D-RUnet, CLAHE

## Abstract

**Purpose:**

The present study is based on evidence indicating a potential correlation between cone-beam CT (CBCT) measurements of tumor size, shape, and the stage of locally advanced rectal cancer. To further investigate this relationship, the study quantitatively assesses the correlation between positioning CT (pCT) and CBCT in the radiomics features of these cancers, and examines their potential for substitution.

**Methods:**

In this study, 103 patients diagnosed with locally advanced rectal cancer and undergoing neoadjuvant chemoradiotherapy were selected as participants. Their CBCT and pCT images were used to divide the participants into two groups: a training set and a validation set, with a 7:3 ratio. An improved conventional 3D-RUNet (CLA-UNet) deep learning model was trained on the training set data and then applied to the validation set. The DSC, HD95 and ASSD were calculated for quantitative evaluation purposes. Then, radiomics features were extracted from 30 patients of the test set.

**Results:**

The experiments demonstrate that, the modified model achieves an average DSC score 0.792 for pCT and 0.672 for CBCT scans. 1037 features were extracted from each patient’s CBCT and pCT images, 73 image features were found to have R values greater than 0.9, including three features related to the staging and prognosis of rectal cancer.

**Conclusion:**

In this study, we proposed an automatic, fast, and consistent method for rectal cancer GTV segmentation for pCT and CBCT scans. The findings of radiomic results indicate that CBCT images have significant research value in the field of radiomics.

## Introduction

The standard-of-care treatment for locally advanced rectal cancer (LARC, T34 or N+) is currently total mesorectal excision (TME) followed by neoadjuvant chemoradiotherapy (nCRT) (1–3). After nCRT, approximately 15%–27% of patients can show a pathologic complete response (pCR) (4, 5). And several prior studies have shown that these patients typically have outstanding long-term outcomes without surgery (6–9). Habr-Gama and colleagues suggested a “wait and see” policy, while Maas and colleagues approach a reasonable solution that could avoid surgery and preserve organs (6, 10). The pCR, however, could only be performed using histopathological analysis of surgically resected specimens. So, it remains a major challenge to develop a non-invasive, validated way to reliably classify pCR patients after chemoradiotherapy.

Tumor segmentation and the subsequent quantitative of rectal cancer in medical images provide valuable information for the analysis of pathologies and prediction of patient outcomes. Numerous studies have shown that image radiomic features extracted from multi-modality imaging techniques, such as CT (11), MRI (12), and PET-CT (13), can be used to predict the treatment response and prognosis of locally advanced rectal cancer. Machine learning models based on CT and MRI image radiomics have also demonstrated good reproducibility and robustness (14, 15). However, these imaging techniques are typically used for disease diagnosis before or after radiotherapy, and are unable to monitor the changes in tumor heterogeneity during the treatment process (16). In contrast, cone-beam CT (CBCT) scans, which are routinely obtained during radiotherapy to examine patient position changes, do not require patients to undergo additional radiation exposure. The features extracted from CBCT may provide valuable information on the changes in tumors during the treatment process without exposing patients to additional radiation hazards. The goal of this study is to examine whether CBCT features can be used for clinical staging or prognosis assessment of tumors by comparing the linear relationship between CBCT and pCT-extracted imaging features.

Precise segmentation of rectal cancer as the mask is particularly important for radiomics extraction and affects the robustness of radiomic features. The current image segmentation methods include manual, semiautomatic, and fully segmentation. The U-Net (17) based models have proven effectiveness over traditional medical segmentation algorithms. However, the 2D U-Net model for segmenting tumors only obtain a single tumor slice in CT scan, while tumors are usually distributed in continuous CT slices (18). To solve the issues, we extend the 2D U-Net to a 3D version with Resnet architecture to capture the inter-slice continuity of the tumor.

## Methods

### Patients

The article under consideration presents a retrospective analysis of 103 patients who underwent neoadjuvant chemoradiotherapy in the Department of Oncology Radiotherapy at the Affiliated Hospital of Xiamen University between January 2019 and October 2020. The study followed the ethical principles outlined in the Helsinki Declaration and its subsequent relevant revisions for all procedures involving human participants. The inclusion criteria for the retrospective analysis were as follows:

1. Biopsy-confirmed primary rectal adenocarcinoma
2. Locally advanced disease (T stage ≥3) prior to treatment
3. No prior receipt of chemoradiotherapy, radiotherapy, or chemotherapy

Only patients who met these criteria were included in the analysis. In compliance with the Helsinki Declaration of 1964 and its later corresponding revisions, all the procedures carried out in this study involving human participants were compliant. CBCT was scanned during the whole treatment period.

### Image acquisition

The CT scans utilized in this study for lesion localization were conducted using a GE LightSpeed device (manufactured by GE Medical System, USA). The scans were performed using parameters of 120 kV tube voltage, 200 mA tube current, a 512×512 reconstruction matrix, and 5mm slice thickness. In addition, CBCT scans were performed using a Truebeam linear accelerator (manufactured by Varian Medical System), with a 512×512 reconstruction matrix and 3mm slice thickness.

### CLA-UNet structure analysis

In this article, we extend the traditional 2D U-Net to 3D U-Net equipped with ResNet architecture to capture the inter-slice continuity of the tumor, and we propose a CLAHE (19) processed U-Net (CLA-UNet) model to further improve the clarity of the anatomy structure, texture, and boundary in the CT image before segmentation. This CLA-UNet designed to accurately segment the lesion area in positioning CT (pCT) and cone-beam CT (CBCT) images of rectal cancer tumor. The CLA-UNet network combines the popular 3D-UNet structure with a residual module (Res-net) to improve the accuracy of tumor location and boundary description, ensuring a precise radiation target area.

**Figure 1:**
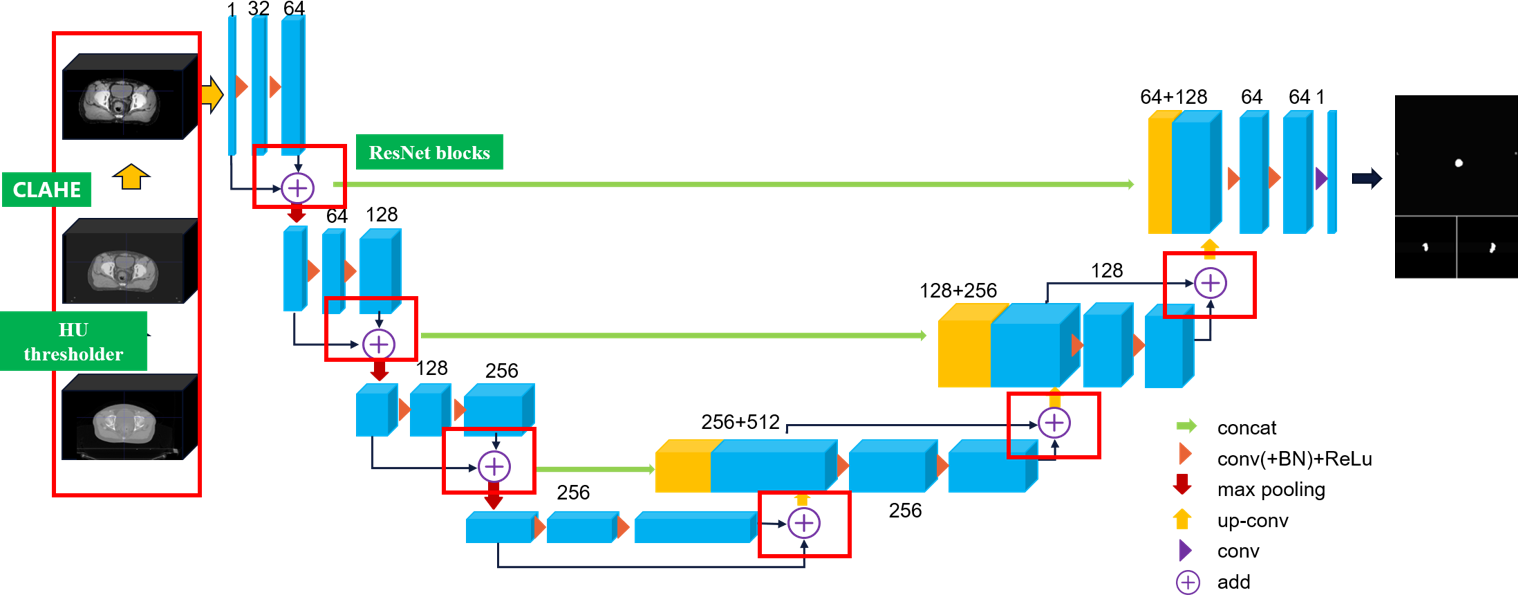
Layers of the proposed CLA-UNet model.

The structure of the CLA-UNet network is illustrated in **Figure 1** of the article. The network is designed to automatically segment the lesion area both in pCT and CBCT images, which also providing valuable information for radiation treatment planning and evaluation.

### CLAHE algorithm processing

Prior to importing the 3D CT data into the 3D-RUNet network for training, a preprocessing step is carried out to crop the CT volume and enhance the image contour. This involves removing any blank areas or areas without drawn target regions, resulting in the cropped CT volume being resized to 256×256×128 voxels using linear interpolation. As rectal tumors are considered soft tissue, the CT value range is restricted to (-200, 300). To focus the network training on information that is relevant to rectal tumors, the 3D CT image is thresholder such that any image values outside the specified range are replaced with corresponding boundary values. To further improve the clarity of the anatomy structure, texture, and boundary in the CT image, the thresholder CT image is processed using the Contrast Limited Adaptive Histogram Equalization (CLAHE) algorithm.

**Figure 2:**
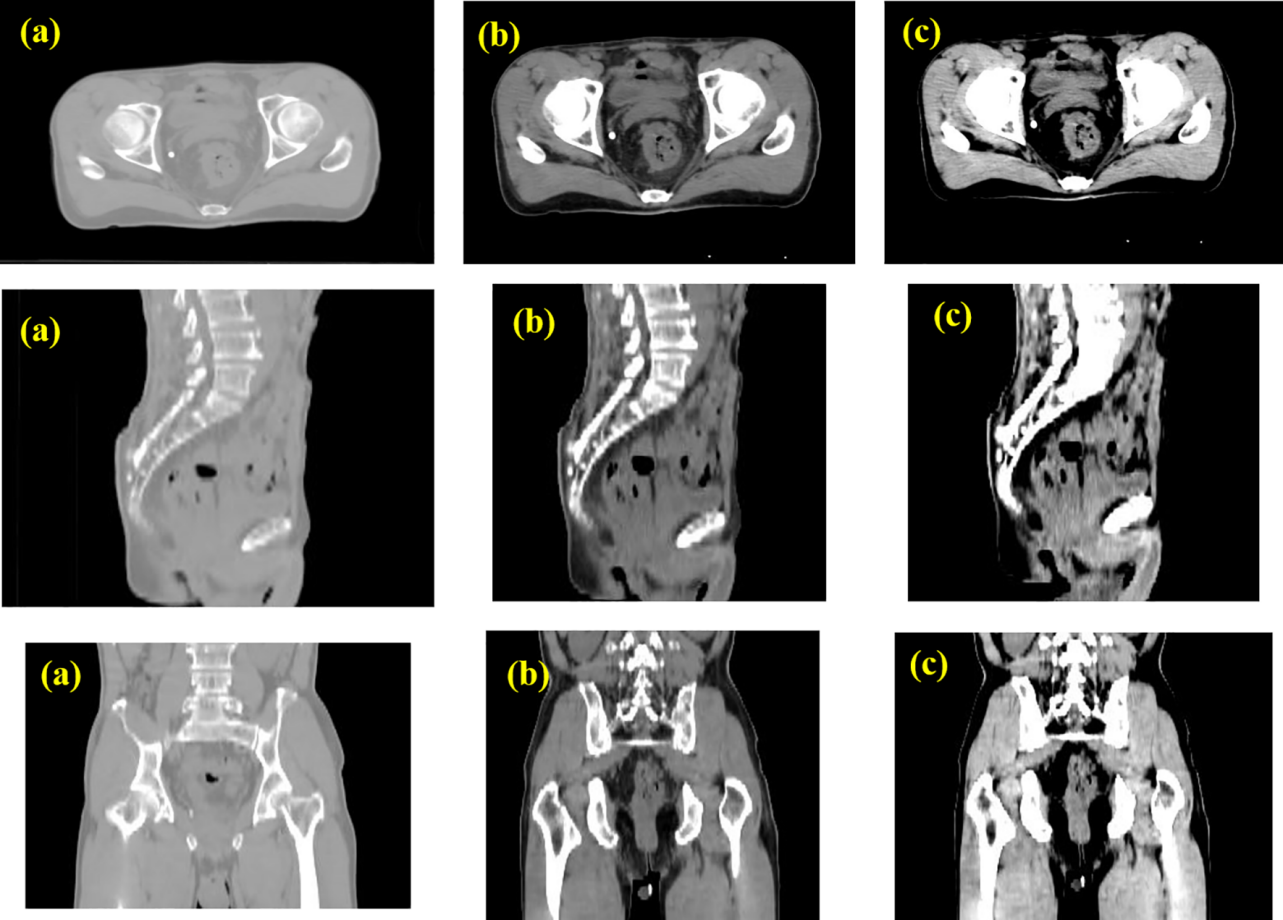
Data pre-processing with CLAHE. **(A)** Original CT image; **(B)** CT image intercepted by threshold with HU=(-200~300); **(C)** CT image transformed by CLAHE.

This results in the rectal structure and boundary becoming clearer and an overall improvement in image quality, as demonstrated in a comparison of the image before and after thresholding and CLAHE processing shown in **Figure 2**.

### Model training and evaluation

The CLA-UNet network is based on the PyTorch kernel platform and the training machine is configured with a Windows 10 operating system and a Quadro P5000 GPU device. The Adam optimizer is used for training with an initial learning rate of 0.0001, and the batch size for network training is set to 2 with a maximum training cycle of 150. This study is trained on a 103 rectal cancer CT dataset provided by the First Affiliated Hospital of Xiamen University, with 70% of the data randomly selected as the training set and 30% as the testing set. The network is trained using the Tversky Loss function, shown in Equation 1.


(1) $$T\left(\alpha ,\beta \right)=\frac{\sum_{i=1}^{N}p_{0i}{ɡ}_{0i}}{\sum_{i=1}^{N}p_{0i}{ɡ}_{0i}+\alpha \sum_{i=1}^{N}p_{0i}{ɡ}_{1i}+\beta \sum_{i=1}^{N}p_{1i}{ɡ}_{0i}}$$

$p_{0i}$ is the probability that the i-th voxel is a tumor, $p_{1i}$ is the probability that the i-th voxel is not a tumor, $g_{0i}$ is 1 if the voxel is abnormal, 0 if it is not abnormal, $g_{1i}$ is the opposite of $g_{0i}$ . Tversky Loss effectively solves the problem of data imbalance by finding a better balance between accuracy and recall.

The model is evaluated using the Dice similarity coefficient (DSC), Hausdorff-95 distance (95% HD), and average symmetric surface distance (ASSD) evaluation metric to compare the segmentation results with those of CBCT scans.

The DSC is defined as follows, shown in Equation 2:


(2) $$\text{DSC}=\frac{2\left|\text{P}\cap \text{G}\right|}{\left|\text{P}\right|+\left|\text{G}\right|}$$

Where the P represents the ground truth, G denotes the prediction results and the $\text{P}\cap^{}\text{G}$ is the intersection of P and G. The range of DSC evaluation is [0,1], and the higher the score is close to 1.0, the more accurate the prediction is. P and G represent the target structure drawn by the physician and the model, respectively.

The HD(A,B) is defined as follows, shown in Equation 3:


(3) $$HD\left(A,B\right)=\text{max}\left(max_{a\in B}\left(min_{b\in B}d\left(a,b\right)\right),max_{b\in B}\left(min_{a\in B}d\left(b,a\right)\right)\right)$$

Where *d* (*a, b*) is the distance between the point a and b.

The ASSD is shown in Equation 4:


(4) $$ASSD=\frac{1}{S\left(A\right)+S\left(B\right)}\left(\sum_{S_{A}\in S\left(A\right)}d\left(S_{A},S\left(B\right)\right)+\sum_{S_{B}\in S\left(B\right)}d\left(S_{B},S\left(A\right)\right)\right)$$

Where *S*(A) represents the surface voxels in set A, and *d*( *S_A_,S*(*B*)) represents the shortest distance from *S_A_* to *S*(*B*).

### Radiomics correlation analysis

The open source radiomics extraction software Pyradiomics 3.0 (https://pyradiomics.readthedocs.io/en/latest/) was used to extract high-throughput features from patient images. In the test group, a total of 30 cases were automatically segmented from pCT and CBCT images using the 3D CLA-UNet model. All the images were filtered by Laplacian of Gaussian (LoG) filter and performed wavelet transformation, so there are four types of images, namely, “Original Images”, “texture Images”, “LoG Images”, and “Wavelet Images”.

After that, the Pearson correlation test was used to analyze the correlation between the image radiomic feature values of pCT and CBCT, if the Pearson correlation coefficient (PCC) R is greater than 0.9, it is considered that the feature value has strong consistency and substitutability in machine learning (14). Pearson correlation coefficient is a method for measuring the similarity of vectors, the range of correlation is [-1, 1], it is defined as the ratio of the covariance and standard deviation of two feature variables, calculated as follows shown in Equation 5:


(5) $$\text{ρ}=\frac{Cov\left(X,Y\right)}{\sigma_{x}\sigma_{y}}=\frac{E\left[\left(X-\mu_{x}\right)\left(Y-\mu_{x}\right)\right]}{\sigma_{x}\sigma_{y}}$$

Among them, X and Y are two different groups of eigenvalue variables, and μ_x and σ_x are mean and standard deviation respectively. This process was implemented using the Pearson algorithm in the R language (R language 3.6).

## Results

### Study population

The radiomics analysis were conducted on a test set of 30 patients, patients’ radiomic characteristics were grouped by LN metastasis and compared in **Supplementary Table 1**. The clinical information includes gender, age, pathology, and clinical-stage information. All the patients received pCRT followed by TME, and group differences were examined.

### Model performance

**Figure 3:**
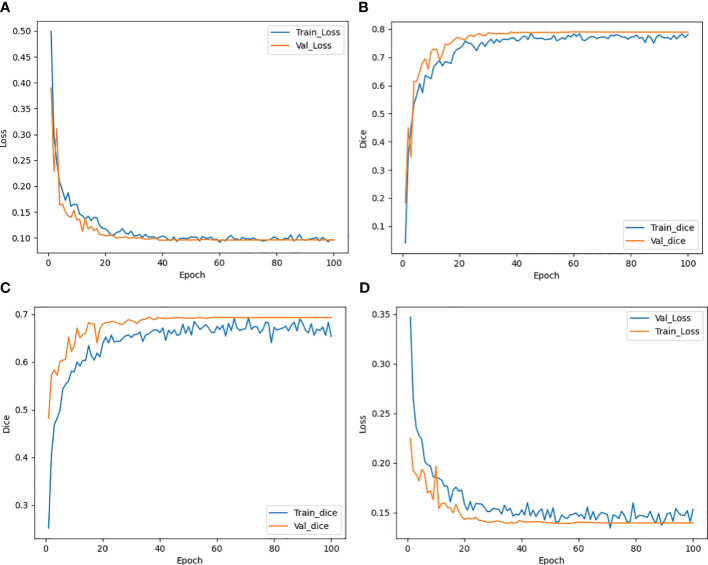
Trend of Loss and Dice value with epoch during training both for CTs **(A, B)** and CBCTs **(C, D)**.

The trend of the average Loss and average Dice values during the CLA-UNet training process is depicted in **Figure 3**. It is evident that as the number of training rounds, also known as epochs, increases, the Loss values (a) on both the training and validation sets rapidly decrease, while the Dice values (b) steadily improve. When the number of epochs reaches 50, the trend stabilizes, with the Loss value reaching close to 0 and the Dice value reaching a stable value around 0.8, details were shown in **Table 1**.

**Table 1:** Comparison results on the test set for the pCT and CBCT scans.

	pCT	CBCT
DSC	0.792 ± 0.056	0.672 ± 0.084
HD95(mm)	15.4 ± 9.5	20.2 ± 12.4
ASSD	4.3 ± 2.1	5.4 ± 2.6

**Figure 4:**
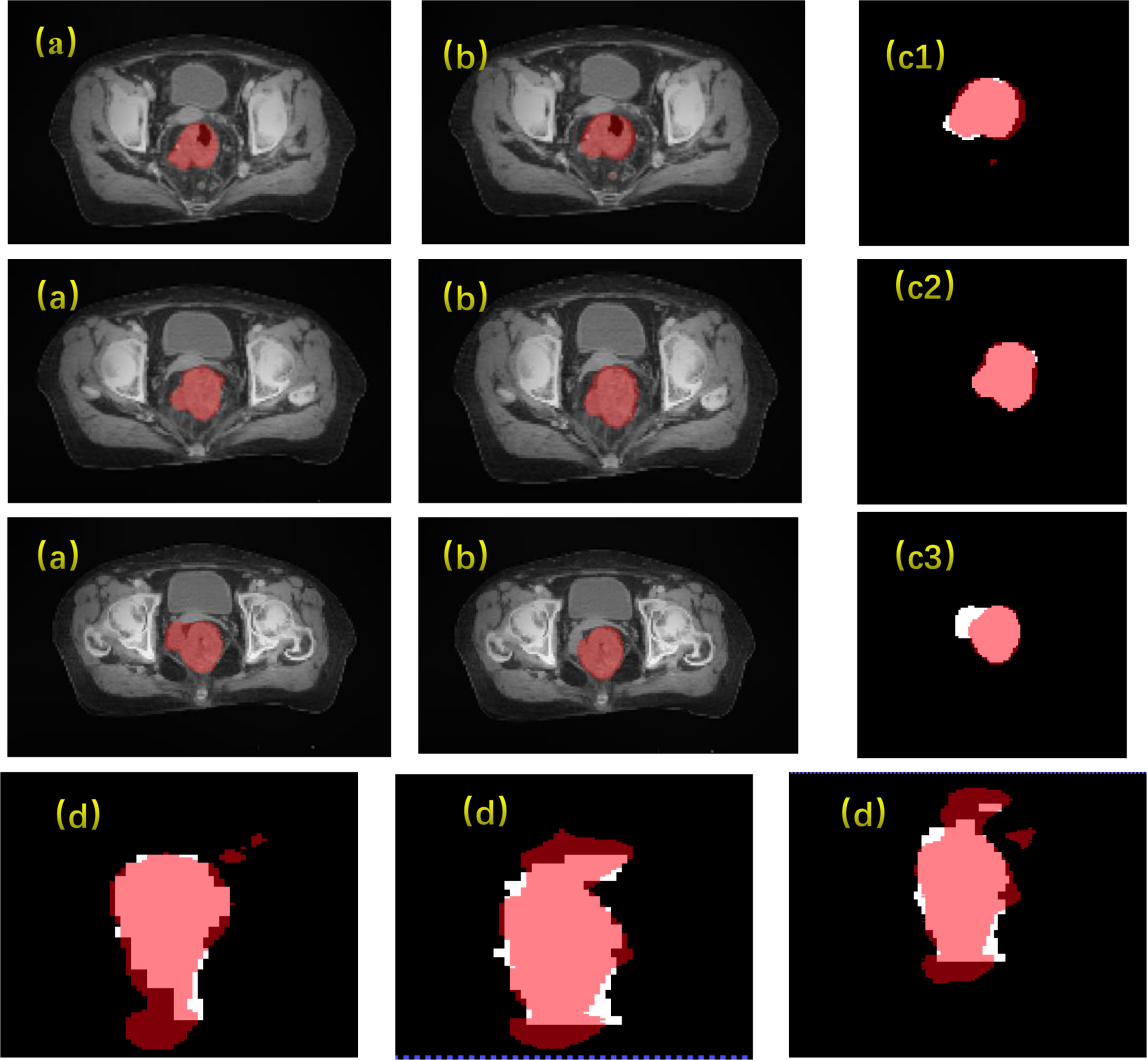
Gross tumor volume contouring with Dice = 0.78. **(A)** manual; **(B)** contouring of CLA-UNet; comparing of segmentations between manual and deep-learning both for transverse **(C)** and coronal **(D)** planes. The red was contoured by CLA-UNet and the white was contoured by manual.

The results of the CLA-UNet network training on 103 samples showed that the network could segment the rectal tumor with good accuracy, details shown in **Figure 4**. As seen in the transverse sections, the performance of the automatic segmentation was satisfactory for the majority of the levels. However, there were some regions, particularly near the cecum and anus, where larger discrepancies were observed between the manual annotations and the machine segmentations. This was likely due to the close proximity of densities in these areas, making it more challenging to distinguish between the different tissues. In such cases, manual annotations by doctors may require additional imaging modalities, such as MRI or PET-CT, or the use of their experience to assist in the outlining process. Despite these limitations, the average Dice score for the CLA-UNet outlining ranged from 0.72 to 0.86, which is generally in line with the clinical requirements.

### Inter-group correlations calculation

**Figure 5:**
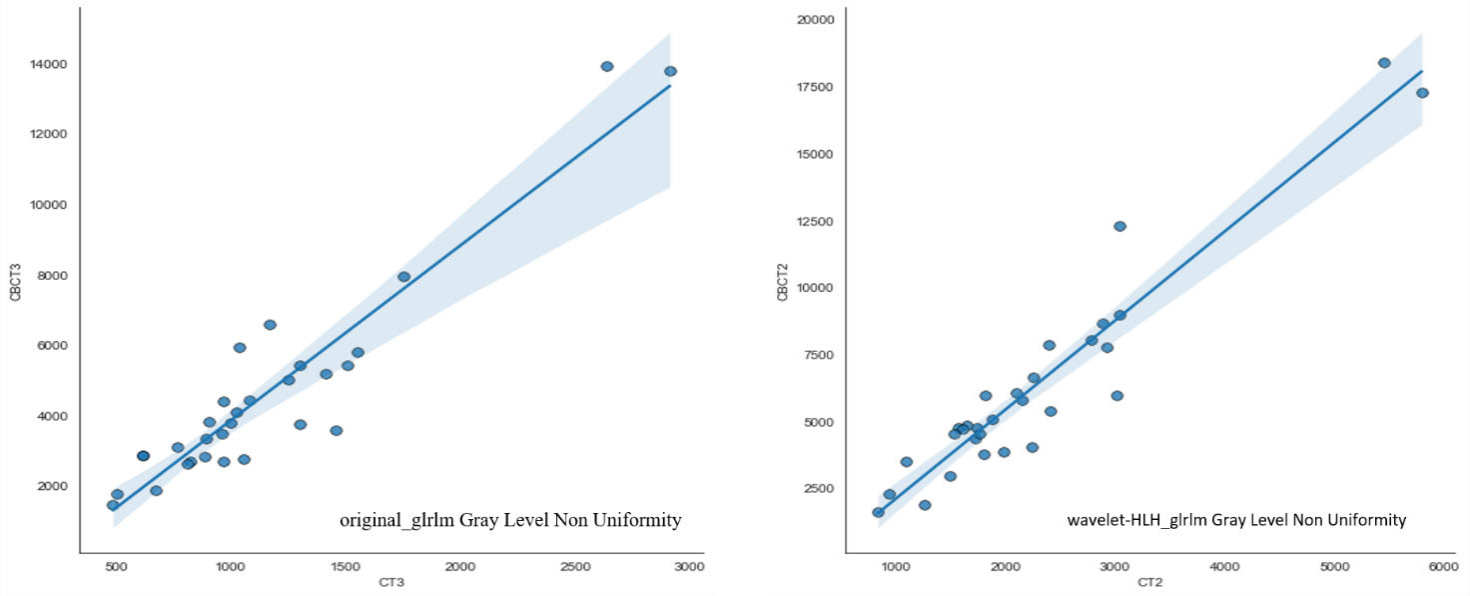
Scatter diagram of features extracted from CBCT and CT scans.

Automatically contouring 30 patient images based on deep learning algorithms to ensure consistency in contouring results. 1037 features were extracted from pCT and CBCT modalities using machine learning algorithms, including shape features (n=14), first-order features (n=19), texture features (n=172), wavelet features (n=728), and loG features (n=104). Pearson’s correlation analysis was used to analyze the correlation of two sets of features, and strong correlated features were extracted. 73 features had Pearson correlation coefficients R greater than 0.9, meaning that these 73 features can be interchangeable. The three features confirmed in previous literature to be related to rectal cancer staging and new adjuvant therapy effectiveness (20), including original first-order Energy, wavelet-HLH_glrlm Gray Level Non Uniformity, and original_glrlm Gray Level Non Uniformity, are included in the strong correlated features. The correlation coefficients R of these three features are 0.9521, 0.9406, and 0.9191, respectively, the data of the subsequent two radiomics were shown in detail in **Figure 5**.

## Discussion

The usage of imaging radiomics in CT scans for rectal cancer diagnosis and prognosis analysis has been well documented in previous studies (21). However, the application of CBCT in this regard has been less explored. In this study, a modified deep learning algorithm, CLA-UNet, was developed to automatically segment the rectal cancer tumor location. With our previous work, we had trained the deep-learning model and used it in our clinical practice. The results indicate that the CLA-UNet model is feasible and time-saving to perform fully automatic segmentation for the rectal tumor both on CBCT and pCT images. To evaluate the accuracy of the 3D mask of the CLA-UNet mode, we compared the coverage of the predicated area with the manual segmentation with an average Dice value, which was 0.792 for pCT and 0.672 for CBCT scans.

Subsequently, imaging radiomic features were extracted and analyzed from both pCT and CBCT scans using machine learning algorithms. The results showed that 73 features had a correlation coefficient (R) greater than 0.9. Our findings also demonstrate that some of the prognostically significant features of radiomics have a strong linear relationship between the pCT and CBCT images based on automatic image segmentation, which indicate a measure of interchangeability between the two scans. These high-correlation features include those previously reported in literature as important indicators for rectal cancer (22, 23). This highlights the potential value of CBCT as an early biomarker for treatment response evaluation (24).

Moreover, high-dimensional features were confirmed in previous literature to be related to rectal cancer staging and new adjuvant therapy effectiveness, in the present study, most of the key features were wavelet features, which are challenging to decipher with the naked eyes. However, high-dimensional features hold more detailed information about the tumor and more sensitive when assessing pCR, as was also demonstrated in recent study (20).

However, there are still some challenges in using CBCT images for radiomics, cause the extracted textural features typically depend on the reconstruction and scanning parameters (25). To be consistent throughout in this study all the CBCTs were resampled into an equal size of 5mm as pCTs, and the influence of slice thickness on the radiomic parameters needs further investigation. Nevertheless, other unknown factors may also influence the consistency evaluation between pCT and CBCT radiomics. Potentially, a radiomics approved reconstruction or corrections could in general improve the consistency and utility of radiomics in medical imaging. Besides this, the detector size of the CBCT has a limited field of view (FOV) that may not be large enough for off-axis patient positions and extensive tumors.

In conclusion, this study provides a preliminary exploration of the correlation between pCT and CBCT imaging radiomics in locally advanced rectal cancer. The CLA-UNet algorithm was successfully applied to segment the rectal tumors, then the correlation between the extracted imaging radiomic features was analyzed. The results showed that radiomic features have a high correlation between pCT and CBCT images, indicating the potential use of CBCT images as an early biomarker for the evaluation of treatment response. However, there are still some limitations in the use of CBCT images. First, the patient sample size was small, a larger sample size test is needed to achieve robust results. Second, the differences in reconstruction algorithms and scan parameters, FOV limitations, and sensitivity to motion artifacts which will influence the consistency evaluation between pCT and CBCT radiomics. Further research is needed to explore the potential applications of CBCT in the diagnosis and prognosis of rectal cancer. Future studies could also focus on multi-center data collection and validation, and on reducing the number of features for clinical predictions.

## Conclusion

In this study, we have presented a modified 3D-UNet segmentation method, CLA-UNet, based on deep learning to automatic segmentation the rectal cancer tumor both for pCTs and CBCTs. Subsequently, radiomic features were extracted and analyzed to find out the inter-group correlation, and the results indicate that some of the prognostically significant features of radiomics have a strong linear relationship between the pCT and CBCT images, which indicate a measure of interchangeability between the two scans.

## Data availability statement

The original contributions presented in the study are included in the article/**Supplementary Material**. Further inquiries can be directed to the corresponding authors.

## Ethics statement

The studies involving humans were approved by the First Affiliated Hospital of Xiamen University medical ethics committee. The studies were conducted in accordance with the local legislation and institutional requirements. Written informed consent for participation was not required from the participants or the participants’ legal guardians/next of kin in accordance with the national legislation and institutional requirements.

## Author contributions

YY: Writing – original draft. GG: Data curation, Software, Writing – review & editing. XG: Conceptualization, Funding acquisition, Writing – review & editing. YH: Formal analysis, Validation, Writing – review & editing. LS: Resources, Visualization, Writing – review & editing. ZK: Funding acquisition, Methodology, Supervision, Writing – original draft. ZZ: Formal analysis, Validation, Writing – review & editing. 
