# Supplementary material for: A study on the radiomic correlation between CBCT and pCT scans based on modified 3D-RUnet image segmentation
Source: Front Oncol. 2024 Feb 22;14:1301710. doi: 10.3389/fonc.2024.1301710 (PMC10921553; doi:10.3389/fonc.2024.1301710)
Supplement: Supplementary file 1 [file DataSheet_1.docx]

Supplementary Material

Unsupervised machine learning based phenogrouping in rectal cancer using radiomics analyses of cone-beam CT images compared to CT images

*Table 1.* *Demographic comparison grouped by Pre-treatment M stage.*

|  |  |  | LN Metastasis (-) | LN Metastasis (+) | P-Value |
| --- | --- | --- | --- | --- | --- |
| Gender, n (%) |  |  | 27 | 3 | 0.783 |
|  | F | 8 (26.7) | 7 (25.9) | 1 (33.3) |  |
|  | M | 22 (73.3) | 20 (74.1) | 2 (66.7) |  |
| age, mean (SD) |  | 56.1 (13.6) | 54.7 (13.4) | 69.0 (8.5) | 0.076 |
| Pre-treatment T stage (%), n (%) |  |  |  |  | 0.587 |
|  | T3 | 18 (60.0) | 17 (63.0) | 1 (33.3) |  |
|  | T4 | 12 (40.0) | 10 (37.0) | 2 (66.7) |  |
| Pre-treatment N stage (%), n (%) |  |  |  |  | 0.008^*^ |
|  | N+ | 9 (30.0) | 9 (33.3) |  |  |
|  | N0 | 8 (26.7) | 8 (29.6) |  |  |
|  | N1 | 5 (16.7) | 5 (18.5) |  |  |
|  | N2 | 5 (16.7) | 4 (14.8) | 1 (33.3) |  |
|  | Nx | 3 (10.0) | 1 (3.7) | 2 (66.7) |  |
| *Note:* Chi-Square were used to compare the differences in categorical variables (Gender, Pre-treatment T stage, Pre-treatment N stage)*.* P value is derived from the univariable association analyses between each of the clinicopathologic variables and LN status.  *Abbreviations:* SD, standard deviation; LN, lymph node  * *P* < 0.05 | | | | | |

Figure1. Clustering of the features extracted from pCT for 30 patients.


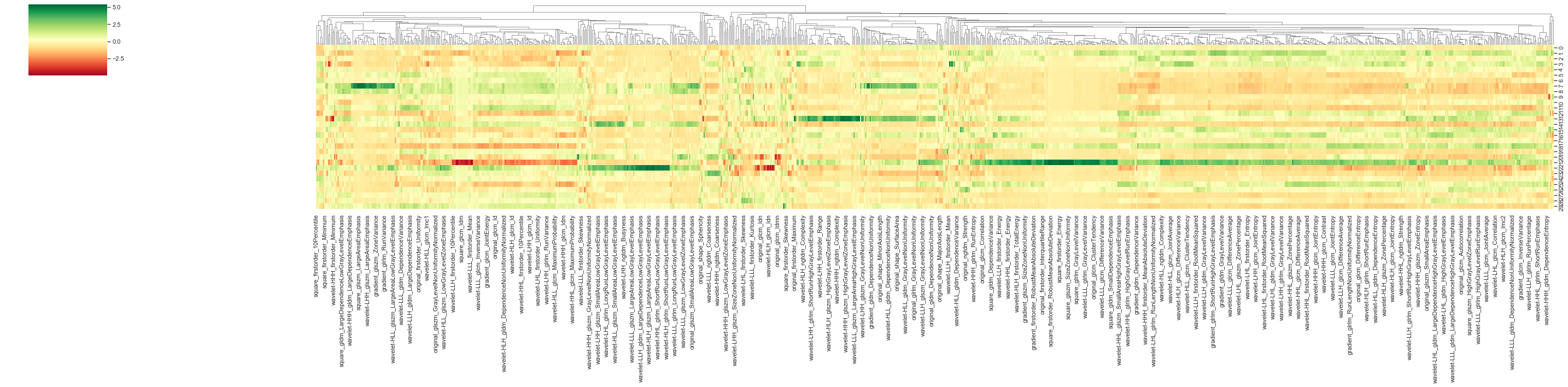


**Scatterplots of interchangeable radiomic features**

Figure 2: Scatterplots of radiomic features derived from pCT and CBCT images for wavelet_HLH glrlmGrayLevelNonUniformity; original_HLH glrlmGrayLevelNonUniformity; original_energy, with Pearson’s r values: 0.9521; 0.9404; 0.9101.

The Figur shows that four same features (original: ‘Energy’; texture: ‘glszm_LAHGLE’;wavelet:‘glszm_LAHGLE’, ‘gldm_LDHGLE’) exacted from pCT and CBCT images, including the previously published 3 prognostic radiomic features

|  | pCT | CBCT |
| --- | --- | --- |
| Features | original_firstorder_Energy | original_firstorder_Energy |
|  | original_glszm_Large Area High Gray Level Emphasis | original_glszm_Large Area High Gray Level Emphasis |
|  | wavelet-LLH_glszm_Large Area High Gray Level Emphasis | wavelet-LLH_glszm_Large Area High Gray Level Emphasis |
|  | wavelet-LLL_gldm_Large Dependence High Gray Level Emphasis | wavelet-LLL_gldm_Large Dependence High Gray Level Emphasis |
|  | square_ngtdm_Coarseness | original_glszm_LargeAreaEmphasis |
|  | square_glszm_Zone Variance |  |

**Linear regression parameters of interchangeable radiomic features**

Table2: First 100 features with highest Person’ r, derived from pCT and CBCT images, Plot numbers correspond to the scatterplots in Figure 2 of Supplementary Material. Abbreviations of feature names are explained at the end of this Supplementary Material.

|  | Pearson’s r | | Fearure name | | |
| --- | --- | --- | --- | --- | --- |
| y = 0.99009 x + -2.66920 | r= | 0.99847 | original | shape | Maximum2DDiameterColumn |
| y = 0.98504 x + 0.12039 | r= | 0.99827 | original | shape | MinorAxisLength |
| y = 1.00137 x + -0.41454 | r= | 0.9982 | original | shape | LeastAxisLength |
| y = 1.00018 x + -2681.37247 | r= | 0.998 | original | shape | MeshVolume |
| y = 0.99896 x + -2819.81972 | r= | 0.998 | original | shape | VoxelVolume |
| y = 0.93880 x + -169.97732 | r= | 0.99607 | original | shape | SurfaceArea |
| y = 0.99696 x + -0.00537 | r= | 0.99568 | original | shape | SurfaceVolumeRatio |
| y = 1.01458 x + 0.00996 | r= | 0.99302 | original | shape | Flatness |
| y = 0.99206 x + -1.23075 | r= | 0.99223 | original | shape | Maximum2DDiameterSlice |
| y = 0.99473 x + -3.08957 | r= | 0.98967 | original | shape | Maximum3DDiameter |
| y = 0.21464 x + 519.37060 | r= | 0.98825 | square | gldm | LargeDependenceHighGrayLevelEmphasis |
| y = 0.98935 x + 0.04265 | r= | 0.98766 | original | shape | Sphericity |
| y = 0.98803 x + -2.72284 | r= | 0.98318 | original | shape | Maximum2DDiameterRow |
| y = 0.99503 x + -2.76150 | r= | 0.98231 | original | shape | MajorAxisLength |
| y = 4.88468 x + -1466.09358 | r= | 0.981 | wavelet-HHH | glrlm | GrayLevelNonUniformity |
| y = 0.99925 x + 0.01996 | r= | 0.98092 | original | shape | Elongation |
| y = 5.16111 x + -5086.41601 | r= | 0.9798 | square | gldm | GrayLevelNonUniformity |
| y = 5.15008 x + -2361.33095 | r= | 0.97877 | wavelet-HHH | gldm | GrayLevelNonUniformity |
| y = 4.00377 x + -131.56273 | r= | 0.97465 | wavelet-HHH | glrlm | RunLengthNonUniformity |
| y = 4.81601 x + -1540.85781 | r= | 0.97389 | wavelet-HHL | glrlm | GrayLevelNonUniformity |
| y = 2.21401 x + -85.45997 | r= | 0.97225 | square | glrlm | GrayLevelNonUniformity |
| y = 0.21694 x + -0.00001 | r= | 0.97196 | wavelet-LLH | ngtdm | Coarseness |
| y = 6.38308 x + -399.20082 | r= | 0.97165 | wavelet-HLL | gldm | DependenceNonUniformity |
| y = 4.68564 x + 111.42790 | r= | 0.97072 | wavelet-LHH | gldm | DependenceNonUniformity |
| y = 4.82384 x + 8.74872 | r= | 0.96894 | log-sigma-1-0-mm-3D | gldm | DependenceNonUniformity |
| y = 5.16423 x + -2761.94317 | r= | 0.96617 | wavelet-HHL | gldm | GrayLevelNonUniformity |
| y = 4.32966 x + -934.72387 | r= | 0.96593 | log-sigma-1-0-mm-3D | glrlm | GrayLevelNonUniformity |
| y = 1.31114 x + 16152959.13314 | r= | 0.96589 | square | firstorder | TotalEnergy |
| y = 6.44586 x + 9150266.75254 | r= | 0.96578 | square | firstorder | Energy |
| y = 6.38114 x + -479.03555 | r= | 0.96494 | wavelet-HHH | gldm | DependenceNonUniformity |
| y = 5.92785 x + -493.19065 | r= | 0.9646 | wavelet-HHL | gldm | DependenceNonUniformity |
| y = 4.98723 x + -3950.64138 | r= | 0.964 | log-sigma-1-0-mm-3D | gldm | GrayLevelNonUniformity |
| y = 0.28750 x + -0.00001 | r= | 0.96317 | log-sigma-1-0-mm-3D | ngtdm | Coarseness |
| y = 0.26129 x + 0.00000 | r= | 0.96316 | wavelet-LHL | ngtdm | Coarseness |
| y = 5.16308 x + -1195.69784 | r= | 0.95493 | wavelet-HLL | glrlm | GrayLevelNonUniformity |
| y = 4.53861 x + 1053.60419 | r= | 0.95369 | wavelet-LLL | glrlm | RunLengthNonUniformity |
| y = 6.98096 x + -695.07178 | r= | 0.95179 | wavelet-HLH | gldm | DependenceNonUniformity |
| y = 0.24988 x + -0.00002 | r= | 0.95076 | wavelet-HLL | ngtdm | Coarseness |
| y = 5.28276 x + -1331.93805 | r= | 0.95013 | wavelet-HLH | glrlm | GrayLevelNonUniformity |
| y = 0.43303 x + -0.00005 | r= | 0.94874 | log-sigma-3-0-mm-3D | ngtdm | Coarseness |
| y = 7.77378 x + 1007.12809 | r= | 0.94829 | square | gldm | DependenceNonUniformity |
| y = 3.13979 x + -256.25742 | r= | 0.94819 | log-sigma-3-0-mm-3D | glrlm | GrayLevelNonUniformity |
| y = 0.28153 x + -0.00003 | r= | 0.94786 | wavelet-LHH | ngtdm | Coarseness |
| y = 38.13750 x + -122504307.64142 | r= | 0.94688 | log-sigma-1-0-mm-3D | glszm | LargeAreaHighGrayLevelEmphasis |
| y = 28.63418 x + -22096109.23987 | r= | 0.94487 | wavelet-HHH | glszm | LargeAreaHighGrayLevelEmphasis |
| y = 5.54022 x + -2007.78649 | r= | 0.94483 | wavelet-HLH | gldm | GrayLevelNonUniformity |
| y = 4.60990 x + -1148.82741 | r= | 0.94157 | wavelet-LHH | glrlm | GrayLevelNonUniformity |
| y = 5.07027 x + -1233.07897 | r= | 0.9406 | original | glrlm | GrayLevelNonUniformity |
| y = 0.28524 x + -0.00003 | r= | 0.94002 | wavelet-HHH | ngtdm | Coarseness |
| y = 4.87805 x + 1672.21850 | r= | 0.93812 | square | glcm | ClusterProminence |
| y = 3.76686 x + -721.58185 | r= | 0.93726 | wavelet-LHL | glrlm | GrayLevelNonUniformity |
| y = 5.39730 x + -2784.12000 | r= | 0.93721 | wavelet-HLL | gldm | GrayLevelNonUniformity |
| y = 5.45095 x + 253.69737 | r= | 0.93578 | wavelet-LHL | gldm | DependenceNonUniformity |
| y = 3.89640 x + 1430.99060 | r= | 0.93372 | wavelet-LLH | glrlm | RunLengthNonUniformity |
| y = 5.93094 x + 73.49680 | r= | 0.93366 | original | gldm | DependenceNonUniformity |
| y = 0.25222 x + -0.00002 | r= | 0.93006 | wavelet-HLH | ngtdm | Coarseness |
| y = 3.66487 x + -3091.81963 | r= | 0.9262 | gradient | gldm | GrayLevelNonUniformity |
| y = 6.02237 x + 1224.20519 | r= | 0.92535 | wavelet-LHL | glrlm | RunLengthNonUniformity |
| y = 2.98414 x + 1471.40401 | r= | 0.92495 | wavelet-HLH | glrlm | RunLengthNonUniformity |
| y = 2.78245 x + 46.31432 | r= | 0.92481 | square | glcm | ClusterShade |
| y = 3.26483 x + -1114.46858 | r= | 0.92202 | wavelet-LHL | gldm | GrayLevelNonUniformity |
| y = 0.30138 x + -0.00003 | r= | 0.92191 | wavelet-HHL | ngtdm | Coarseness |
| y = 3.76906 x + 608.58017 | r= | 0.92086 | wavelet-HHL | glrlm | RunLengthNonUniformity |
| y = 2.35916 x + 667754302.47334 | r= | 0.92026 | wavelet-LLL | firstorder | Energy |
| y = 0.47934 x + 1185602766.81108 | r= | 0.91926 | wavelet-LLL | firstorder | TotalEnergy |
| y = 14.20729 x + -2751647.86359 | r= | 0.91766 | wavelet-HHL | glszm | ZoneVariance |
| y = 0.49480 x + 670598.80113 | r= | 0.91685 | wavelet-LLL | glcm | ClusterProminence |
| y = 4.64829 x + -1969.03357 | r= | 0.91669 | wavelet-LHH | gldm | GrayLevelNonUniformity |
| y = 4.50786 x + -480.60636 | r= | 0.91066 | wavelet-LLL | glrlm | GrayLevelNonUniformity |
| y = 2.21913 x + 91515094.85197 | r= | 0.91012 | original | firstorder | Energy |
| y = 1.27072 x + 0.20401 | r= | 0.90972 | square | glcm | Autocorrelation |
| y = 0.45087 x + 163410641.18549 | r= | 0.90885 | original | firstorder | TotalEnergy |
| y = 1.41023 x + 1.67086 | r= | 0.90037 | square | glcm | ClusterTendency |

**Radiomics Feature Extraction**

Since the intensity values of MR images distribute widely, we used z-score normalization to make the image intensities have the properties of a standard normal distribution with $\mu=1$ and $\sigma=0$, where $\mu$ is the mean value of the images, and $\sigma$ is the standard deviation. The normalized values (also called z scores) of the image intensities (*x*) were calculated as follows:

$$z= \frac{x-\mu}{\sigma}$$

After z-score normalization of image pixel intensities, a total of 563 quantitative imaging features including 4 statistical features, 43 voxel-intensity computational features and 516 wavelet features, were extracted respectively for T2 images and ADC maps of each scan using corresponding ROIs.

**(1) Statistical features**

The following 4 statistical features were extracted from the ROI of each patient’s T2 images and ADC maps for each scan: IntensityMax, IntensityMin, IntensityAve, IntensityStd, IntensityVar, Skewness, and Kurtosis. IntensityMax, IntensityMin, IntensityAve, and IntensityStd were the maximum, minimum, average, and standard deviation intensity value of the ROI, respectively.

Let VI(i,j,k) be the intensity of the ROI.

1. **IntensityMax:** The maximum intensity value of **VI.**
2. **IntensityMin:** The minimum intensity value of **VI**.
3. **IntensityAve:** The average intensity value of **VI**.
4. **IntensityStd:**

$$IntensityStd=(\frac{1}{L*W*H-1}\sum_{i=1}^{L} \sum_{j=1}^{W} \sum_{k=1}^{H} (\mathrm{VI}(\mathrm{i},\mathrm{j},\mathrm{k})-IntensityAve)^{2})^{1/2}$$

**(2)** **Voxel-intensity computational features**

Forty-three voxel-intensity computational based features including the first order statistic texture features, second order statistic texture features, and higher order statistic texture features were extracted. Three first order statistic texture features including: Variance, Skewness, and Kurtosis were extracted to describe the intensity histogram distribution of the tumor region. Nine second order statistic texture features could be calculated from the Gray Level Co-occurrence Matrix (GLCM). Thirty-one high order statistic texture features were calculated from the Gray Level Size Zone Matrix (GLSZM), Gray Level Run Length Matrix (GLRLM), and Neighborhood Gray Tone Difference Matrix (NGTDM). All of the GLCM, GLSZM, GLRLM, and NGTDM based texture feature were calculated using a 2D analysis and then averaged for all slices within the three-dimensional tumor volume.

*First-order statistical features*

First-order statistical features described the distribution of voxel intensities of the tumor region. Let **P** represent the 3-D image histogram distribution. The following first-order statistical features were extracted:

1. **Variance:**

$$\sqrt{\sum_{j=1}^{N_{g}} P(\mathrm{j})*(\mathrm{j}-\sum_{i=1}^{N_{g}} P(\mathrm{i})*\mathrm{i})^{2}}$$

1. **Skewness:**

$$\sqrt{\sum_{j=1}^{N_{g}} P(\mathrm{j})*(\mathrm{j}-\sum_{i=1}^{N_{g}} P(\mathrm{i})*\mathrm{i})^{3}}$$

1. **Kurtosis:**

$$\sqrt{\sum_{j=1}^{N_{g}} P(\mathrm{j})*(\mathrm{j}-\sum_{i=1}^{N_{g}} P(\mathrm{i})*\mathrm{i})^{4}}$$

*Gray-Level Co-Occurrence Matrix based features (GLCM)*

GLCM based features were second-order statistical texture features, which are defined as a matrix *M*(*i, j; δ, θ*) to indicate the relative frequency with intensity values of pixels (*i* and *j*) at the distance of *δ* in direction *θ*.

Let:

*M*(*i, j*) be the co-occurrence matrix for an arbitrary *δ* and *θ*,

*N_g_* be the number of discrete intensity levels in the images,

*μ* be the mean of *M*(*i, j*),

$m_{x}(\mathrm{i})=\sum_{j=1}^{N_{g}} M(\mathrm{i},\mathrm{j})$ be the marginal row probabilities,

$m_{y}(\mathrm{i})=\sum_{i=1}^{N_{g}} M(\mathrm{i},\mathrm{j})$ be the marginal column probabilities, and

*μ_x_* be the mean of *m_x_*.

1. **Energy:**

$$\sum_{i=1}^{N_{g}} \sum_{j=1}^{N_{g}} [\mathrm{M}(\mathrm{i},\mathrm{j})]^{2}$$

1. **Contrast:**

$$\sum_{i=1}^{N_{g}} \sum_{j=1}^{N_{g}} (\mathrm{i}-\mathrm{j})^{2}*M(\mathrm{i},\mathrm{j})$$

1. **Entropy:**

$$\sum_{i=1}^{N_{g}} \sum_{j=1}^{N_{g}} M(\mathrm{i},\mathrm{j})*\log_{2}M(\mathrm{i},\mathrm{j})$$

1. **Homogeneity I:**

$$\sum_{i=1}^{N_{g}} \sum_{j=1}^{N_{g}} \frac{M(\mathrm{i},\mathrm{j})}{1+|\mathrm{i}-\mathrm{j}|}$$

1. **Correlation:**

$$\frac{\sum_{i=1}^{N_{g}} \sum_{j=1}^{N_{g}} ijM(\mathrm{i},\mathrm{j})-\mu_{i}(\mathrm{i})\mu_{j}(\mathrm{j})}{\sigma_{x}(\mathrm{i})\sigma_{y}(\mathrm{j})}$$

1. **Variance:**

$$\sum_{i=1}^{N_{g}} \sum_{j=1}^{N_{g}} (\mathrm{i}-\mu)^{2}M(\mathrm{i},\mathrm{j})$$

1. **Sum Average:**

$$\sum_{i=2}^{2N_{g}} iM_{x+y}(\mathrm{i})$$

1. **Dissimilarity:**

$$\sum_{i=1}^{N_{g}} \sum_{j=1}^{N_{g}} |\mathrm{i}-\mathrm{j}|\mathrm{M}(\mathrm{i},\mathrm{j})$$

1. **Inverse Difference Moment:**

$$\sum_{i=1}^{N_{g}} \sum_{j=1}^{N_{g}} \frac{\mathrm{M}(\mathrm{i},\mathrm{j})}{1+(\frac{|\mathrm{i}-\mathrm{j}|^{2}}{N^{2}})}$$

*Gray Level Run Length Matrix based features (GLRLM)*

GLRLM based features were high-order statistical texture feature, which were defined as *P*(*i, j; θ*) to indicate the number of times j and gray level i appear consecutively in the direction *θ*.

Let:

*P*(*i, j; θ*) be the run-length matrix *P* for a direction *θ*,

*N_g_* be the number of discrete intensity values,

*N_r_* be the number of different run lengths, and

*N_p_* be the number of voxels in the ROI.

1. **Short Run Emphasis (SRE):**

$$\frac{\sum_{i=1}^{N_{g}} \sum_{j=1}^{N_{r}} [\frac{P(\mathrm{i},\mathrm{j};\theta)}{j^{2}}]}{\sum_{i=1}^{N_{g}} \sum_{j=1}^{N_{r}} P(\mathrm{i},\mathrm{j};\theta)}$$

1. **Long Run Emphasis (LRE):**

$$\frac{\sum_{i=1}^{N_{g}} \sum_{j=1}^{N_{r}} j^{2}P(\mathrm{i},\mathrm{j};\theta)}{\sum_{i=1}^{N_{g}} \sum_{j=1}^{N_{r}} P(\mathrm{i},\mathrm{j};\theta)}$$

1. **Gray-Level Nonuniformity (GLN):**

$$\frac{\sum_{i=1}^{N_{g}} [\sum_{j=1}^{N_{r}} P(\mathrm{i},\mathrm{j};\theta)]^{2}}{\sum_{i=1}^{N_{g}} \sum_{j=1}^{N_{r}} P(\mathrm{i},\mathrm{j};\theta)}$$

1. **Run-Length Nonuniformity (RLN):**

$$\frac{\sum_{j=1}^{N_{r}} [\sum_{i=1}^{N_{g}} P(\mathrm{i},\mathrm{j};\theta)]^{2}}{\sum_{i=1}^{N_{g}} \sum_{j=1}^{N_{r}} P(\mathrm{i},\mathrm{j};\theta)}$$

1. **Run Percentage (RP):**

$$\sum_{i=1}^{N_{g}} \sum_{j=1}^{N_{r}} \frac{P(\mathrm{i},\mathrm{j};\theta)}{N_{p}}$$

1. **Low Gray-Level Run Emphasis (LGRE):**

$$\frac{\sum_{i=1}^{N_{g}} \sum_{j=1}^{N_{r}} [\frac{P(\mathrm{i},\mathrm{j};\theta)}{i^{2}}]}{\sum_{i=1}^{N_{g}} \sum_{j=1}^{N_{r}} P(\mathrm{i},\mathrm{j};\theta)}$$

1. **High Gray-Level Run Emphasis (HGRE):**

$$\frac{\sum_{i=1}^{N_{g}} \sum_{j=1}^{N_{r}} i^{2}P(\mathrm{i},\mathrm{j};\theta)}{\sum_{i=1}^{N_{g}} \sum_{j=1}^{N_{r}} P(\mathrm{i},\mathrm{j};\theta)}$$

1. **Short Run Low Gray-Level Emphasis (SRLGE):**

$$\frac{\sum_{i=1}^{N_{g}} \sum_{j=1}^{N_{r}} \frac{P(\mathrm{i},\mathrm{j};\theta)}{i^{2}j^{2}}}{\sum_{i=1}^{N_{g}} \sum_{j=1}^{N_{r}} P(\mathrm{i},\mathrm{j};\theta)}$$

1. **Short Run High Gray-Level Emphasis (SRHGE):**

$$\frac{\sum_{i=1}^{N_{g}} \sum_{j=1}^{N_{r}} \frac{i^{2}P(\mathrm{i},\mathrm{j};\theta)}{j^{2}}}{\sum_{i=1}^{N_{g}} \sum_{j=1}^{N_{r}} P(\mathrm{i},\mathrm{j};\theta)}$$

1. **Long Run Low Gray-Level Emphasis (LRLGE):**

$$\frac{\sum_{i=1}^{N_{g}} \sum_{j=1}^{N_{r}} \frac{j^{2}P(\mathrm{i},\mathrm{j};\theta)}{i^{2}}}{\sum_{i=1}^{N_{g}} \sum_{j=1}^{N_{r}} P(\mathrm{i},\mathrm{j};\theta)}$$

1. **Long Run High Gray-Level Emphasis (LRHGE):**

$$\frac{\sum_{i=1}^{N_{g}} \sum_{j=1}^{N_{r}} i^{2}j^{2}P(\mathrm{i},\mathrm{j};\theta)}{\sum_{i=1}^{N_{g}} \sum_{j=1}^{N_{r}} P(\mathrm{i},\mathrm{j};\theta)}$$

1. **Gray-Level Variance (GLV):**

$$\sum_{i=1}^{N_{g}} \sum_{j=1}^{N_{r}} \{\mathrm{iP}(\mathrm{i},\mathrm{j};\theta)-\frac{\sum_{i=1}^{N_{g}} i[\sum_{j=1}^{N_{r}} P(\mathrm{i},\mathrm{j};\theta)]}{N_{g}N_{r}}\}$$

1. **Run-Length Variance (RLV):**

$$\sum_{i=1}^{N_{g}} \sum_{j=1}^{N_{r}} \{\mathrm{jP}(\mathrm{i},\mathrm{j};\theta)-\frac{\sum_{i=1}^{N_{g}} i[\sum_{j=1}^{N_{r}} P(\mathrm{i},\mathrm{j};\theta)]}{N_{g}N_{r}}\}$$

*Gray Level Size Zone Matrix based features (GLSZM)*

GLSZM based features were high-order statistical texture features, which were defined as *P*(*i, j*) to indicate the areas of size j and gray level i.

Let:

*P*(*i, j*) be the size zone of matrix *P*,

*N_g_* be the number of discrete intensity values,

*N_r_* be the number of different areas sizes,

*N_p_* be the number of voxels in the ROI.

1. **Small Zone Emphasis (SZE):**

$$\frac{\sum_{i=1}^{N_{g}} \sum_{j=1}^{N_{r}} [\frac{P(\mathrm{i},\mathrm{j})}{j^{2}}]}{\sum_{i=1}^{N_{g}} \sum_{j=1}^{N_{r}} P(\mathrm{i},\mathrm{j})}$$

1. **Large Zone Emphasis (LZE):**

$$\frac{\sum_{i=1}^{N_{g}} \sum_{j=1}^{N_{r}} j^{2}P(\mathrm{i},\mathrm{j})}{\sum_{i=1}^{N_{g}} \sum_{j=1}^{N_{r}} P(\mathrm{i},\mathrm{j})}$$

1. **Gray-Level Nonuniformity (GLN):**

$$\frac{\sum_{i=1}^{N_{g}} [\sum_{j=1}^{N_{r}} P(\mathrm{i},\mathrm{j})]^{2}}{\sum_{i=1}^{N_{g}} \sum_{j=1}^{N_{r}} P(\mathrm{i},\mathrm{j})}$$

1. **Zone-Size Nonuniformity (ZSN):**

$$\frac{\sum_{j=1}^{N_{r}} [\sum_{i=1}^{N_{g}} P(\mathrm{i},\mathrm{j})]^{2}}{\sum_{i=1}^{N_{g}} \sum_{j=1}^{N_{r}} P(\mathrm{i},\mathrm{j})}$$

1. **Zone Percentage (ZP):**

$$\sum_{i=1}^{N_{g}} \sum_{j=1}^{N_{r}} \frac{P(\mathrm{i},\mathrm{j})}{N_{p}}$$

1. **Low Gray-Level Zone Emphasis (LGZE):**

$$\frac{\sum_{i=1}^{N_{g}} \sum_{j=1}^{N_{r}} [\frac{P(\mathrm{i},\mathrm{j})}{i^{2}}]}{\sum_{i=1}^{N_{g}} \sum_{j=1}^{N_{r}} P(\mathrm{i},\mathrm{j})}$$

1. **High Gray-Level Zone Emphasis (HGZE):**

$$\frac{\sum_{i=1}^{N_{g}} \sum_{j=1}^{N_{r}} i^{2}P(\mathrm{i},\mathrm{j})}{\sum_{i=1}^{N_{g}} \sum_{j=1}^{N_{r}} P(\mathrm{i},\mathrm{j})}$$

1. **Small Zone Low Gray-Level Emphasis (SZLGE):**

$$\frac{\sum_{i=1}^{N_{g}} \sum_{j=1}^{N_{r}} \frac{P(\mathrm{i},\mathrm{j})}{i^{2}j^{2}}}{\sum_{i=1}^{N_{g}} \sum_{j=1}^{N_{r}} P(\mathrm{i},\mathrm{j})}$$

1. **Small Zone High Gray-Level Emphasis (SZHGE):**

$$\frac{\sum_{i=1}^{N_{g}} \sum_{j=1}^{N_{r}} \frac{i^{2}P(\mathrm{i},\mathrm{j})}{j^{2}}}{\sum_{i=1}^{N_{g}} \sum_{j=1}^{N_{r}} P(\mathrm{i},\mathrm{j})}$$

1. **Large Zone Low Gray-Level Emphasis (LZLGE):**

$$\frac{\sum_{i=1}^{N_{g}} \sum_{j=1}^{N_{r}} \frac{j^{2}P(\mathrm{i},\mathrm{j})}{i^{2}}}{\sum_{i=1}^{N_{g}} \sum_{j=1}^{N_{r}} P(\mathrm{i},\mathrm{j})}$$

1. **Large Zone High Gray-Level Emphasis (LZHGE):**

$$\frac{\sum_{i=1}^{N_{g}} \sum_{j=1}^{N_{r}} i^{2}j^{2}P(\mathrm{i},\mathrm{j})}{\sum_{i=1}^{N_{g}} \sum_{j=1}^{N_{r}} P(\mathrm{i},\mathrm{j})}$$

1. **Gray-Level Variance (GLV):**

$$\sum_{i=1}^{N_{g}} \sum_{j=1}^{N_{r}} \{\mathrm{iP}(\mathrm{i},\mathrm{j})-\frac{\sum_{i=1}^{N_{g}} i[\sum_{j=1}^{N_{r}} P(\mathrm{i},\mathrm{j})]}{N_{g}N_{r}}\}$$

1. **Zone-Size Variance (ZSV):**

$$\sum_{i=1}^{N_{g}} \sum_{j=1}^{N_{r}} \{\mathrm{jP}(\mathrm{i},\mathrm{j})-\frac{\sum_{i=1}^{N_{g}} i[\sum_{j=1}^{N_{r}} P(\mathrm{i},\mathrm{j})]}{N_{g}N_{r}}\}$$

*Neighborhood Gray Tone Difference Matrix based features (NGTDM)*

NGTDM based features were high-order statistical texture features, which were defined as *S(i)* to indicate the sum of the absolute value between gray intensity level i and it’s neighbors’ average intensity.

Let:

*S(i)* be the sum of absolute value between gray intensity level i and its neighbors’ average intensity,

*C(i)* be the number of voxels with the gray intensity level I,

*N_g_* be the number of discrete intensity values.

1. **Coarseness:**

$$\frac{1}{\varepsilon+\sum_{i=1}^{N_{g}} \frac{C(\mathrm{i})\mathrm{S}(\mathrm{i})}{\sum_{i=1}^{N_{g}} C(\mathrm{i})}}$$

1. **Contrast:**

$$\frac{\sum_{i=1}^{N_{g}} \sum_{j=1}^{N_{g}} C(\mathrm{i})\mathrm{C}(\mathrm{j})(\mathrm{i}-\mathrm{j})^{2}}{(\sum_{i=1}^{N_{g}} C(\mathrm{i}))^{2}}*\sum_{i=1}^{N_{g}} S(\mathrm{i})*\frac{1}{N_{g}(\mathrm{N}_{g}-1)\sum_{i=1}^{N_{g}} C(\mathrm{i})}$$

1. **Busyness:**

$$\frac{\sum_{i=1}^{N_{g}} C(\mathrm{i})\mathrm{S}(\mathrm{i})}{\sum_{i=1}^{N_{g}} \sum_{j=1}^{N_{g}} (\mathrm{iC}(\mathrm{i})-\mathrm{jC}(\mathrm{j}))}, C(\mathrm{i})\neq0,\mathrm{C}(\mathrm{j})\neq0$$

1. **Complexity:**

$$\frac{1}{(\sum_{i=1}^{N_{g}} C(\mathrm{i}))^{2}}*\sum_{i=1}^{N_{g}} \sum_{j=1}^{N_{g}} \frac{|\mathrm{i}-\mathrm{j}|(\mathrm{C}(\mathrm{i})\mathrm{S}(\mathrm{i})+\mathrm{C}(\mathrm{j})\mathrm{S}(\mathrm{j}))}{(\mathrm{C}(\mathrm{i})+\mathrm{C}(\mathrm{j}))}, C(\mathrm{i})\neq0,\mathrm{C}(\mathrm{j})\neq0$$

1. **Strength:**

$$\frac{\sum_{i=1}^{N_{g}} \sum_{j=1}^{N_{g}} (\mathrm{C}(\mathrm{i})-\mathrm{C}(\mathrm{j}))*(\mathrm{i}-\mathrm{j})^{2}}{\sum_{i=1}^{N_{g}} C(\mathrm{i})*\sum_{i=1}^{N_{g}} S(\mathrm{i})}, C(\mathrm{i})\neq0,\mathrm{C}(\mathrm{j})\neq0$$

**(3) Wavelet features: first order statistical, second order statistical, and high order statistical texture of a wavelet filtered image.**

A total of 516 wavelet based features were extracted. By using the Gabor-bank wavelet, we obtained four different scales and eight different orientations filtered images. These wavelet-based features were computed on the filtered images. The original image was filtered by a two-dimensional Gabor filter defined as:

In this study, four frequency scales $f=\frac{0.25}{(\sqrt{2})^{i-1}}, i=1,2,3,4$ and eight orientations$\theta=\frac{j-1}{8}\pi, j=1,2,....,8$ were used. After filtering, 32 filtered images were detected. For each frequency scale, the filtered images of 8 different orientations were summed, and then four new images were generated. For each orientation, the filtered images of 4 different scales were summed, and then 8 new images were generated. After these operations, 12 filtered images were generated. For each image, the first order statistical, second order statistical, and high order statistical texture features were computed.


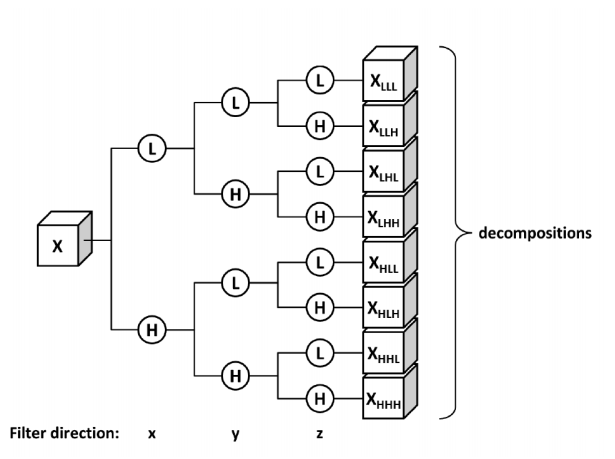


**Laplacian of Gaussian features**

The Laplacian of an image brings out areas of rapid intensity change and is usually used for edge detection. A Gaussian filter is applied prior to the Laplacian to smooth the image and reduce noise. Textural properties representing features of different degrees of coarseness can then be calculated. The equation of a LoG with a 2D kernel:

$$LoG\left( x,y \right)=-\frac{1}{\pi\sigma^{4}}[1-\frac{x^{2}+y^{2}}{2\sigma^{2}}]e^{-\frac{x^{2}+y^{2}}{2\sigma^{2}}}$$

Texture size (fine to coarse) is highlighted by modifying the Gaussian radius parameter 𝜎 (e.g., from 0.5 mm to 5mm, with 0.5 mm increments). Each value of 𝜎 provides a filtered image. First-order gray-level statistics (described earlier) are determined for each filtered image, as well as for only the positive part of each filtered image.
